# Impaired CD4 T Cell Memory Response to *Streptococcus pneumoniae* Precedes CD4 T Cell Depletion in HIV-Infected Malawian Adults

**DOI:** 10.1371/journal.pone.0025610

**Published:** 2011-09-27

**Authors:** Sarah J. Glennie, Enoch Sepako, David Mzinza, Visopo Harawa, David J. C. Miles, Kondwani C. Jambo, Stephen B. Gordon, Neil A. Williams, Robert S. Heyderman

**Affiliations:** 1 Malawi-Liverpool-Wellcome Trust Clinical Research Programme, University of Malawi College of Medicine, Blantyre, Malawi; 2 South African Tuberculosis Vaccine Institute, Institute of Infectious Disease and Molecular Medicine, University of Cape Town, Cape Town, South Africa; 3 Respiratory Infection Group, Liverpool School of Tropical Medicine, Liverpool, United Kingdom; 4 Cellular and Molecular Medicine, School of Medical Sciences, University of Bristol, Bristol, United Kingdom; MRC National Institute for Medical Research, United Kingdom

## Abstract

**Objective:**

Invasive pneumococcal disease (IPD) is a leading cause of morbidity and mortality in HIV-infected African adults. CD4 T cell depletion may partially explain this high disease burden but those with relatively preserved T cell numbers are still at increased risk of IPD. This study evaluated the extent of pneumococcal-specific T cell memory dysfunction in asymptomatic HIV infection early on in the evolution of the disease.

**Methods:**

Peripheral blood mononuclear cells were isolated from asymptomatic HIV-infected and HIV-uninfected Malawian adults and stained to characterize the underlying degree of CD4 T cell immune activation, senescence and regulation. Pneumococcal-specific T cell proliferation, IFN-γ, IL-17 production and CD154 expression was assessed using flow cytometry and ELISpot.

**Results:**

We find that in asymptomatic HIV-infected Malawian adults, there is considerable immune disruption with an increase in activated and senescent CD4^+^CD38^+^PD-1^+^ and CD4^+^CD25^high^Foxp3^+^ Treg cells. In the context of high pneumococcal exposure and therefore immune stimulation, show a failure in pneumococcal-specific memory T cell proliferation, skewing of T cell cytokine production with preservation of interleukin-17 but decreased interferon-gamma responses, and failure of activated T cells to express the co-stimulatory molecule CD154.

**Conclusion:**

Asymptomatic HIV-infected Malawian adults show early signs of pneumococcal- specific immune dysregulation with a shift in the balance of CD4 memory, T helper 17 cells and Treg. Together these data offer a mechanistic understanding of how antigen-specific T cell dysfunction occurs prior to T cell depletion and may explain the early susceptibility to IPD in those with relatively preserved CD4 T cell numbers.

## Introduction

Worldwide, *Streptococcus pneumoniae* is a leading cause of pneumonia, bacteremia and meningitis, resulting in approximately 1.1 million deaths annually[1], [2]. Sub-Saharan Africa has a disproportionate share of this burden of invasive pneumococcal disease (IPD), which in 85–90% of adults is HIV-associated[3]. Even in the early stages, when CD4 counts are relatively preserved, HIV-infected individuals are up to 20 times more likely to develop IPD[4], [5].

In healthy people, the predominance of pneumococcal disease in children under 5 years and the fall in the prevalence of nasopharyngeal carriage in early adulthood suggest that there is acquisition of natural anti-pneumococcal immunity with age. Previously, protective immunity to the pneumococcus in humans was thought to be largely mediated by antibodies to the pneumococcal polysaccharide capsule[6], however, we and others have described naturally acquired B and T cell immunity to a variety of pneumococcal proteins[7], [8]. Studies of colonization, antibody acquisition and the relationship with otitis media suggest that naturally-induced antibodies to pneumococcal protein antigens are protective against disease[9], [10]. Pneumococcal specific T cell responses promote the maturation of these B cell responses. In addition, recent data suggests that T-helper 1 (Th1) and Th17 cells may facilitate the clearance of pneumococcal colonization within the nasopharynx[11], [12] and that Th1 cells control the multiplication of bacteria following dissemination[13], [14].

In HIV infection, it is now widely recognized that the decline of CD4 T cells is not equally distributed throughout lymphoid tissues and that accelerated depletion occurs at sites of “persistent inflammation” such as mucosal surfaces[15]. Given that *Streptococcus pneumoniae* is found in the nasopharynx of up to 28% of HIV infected African adults[16], it is logical that depletion of pneumococcal-specific mucosal T cells predispose HIV-infected individuals to invasive disease. However, the observation that individuals with relatively preserved peripheral CD4 T cells are still susceptible to IPD suggests that either this compromised T cell immunity is largely compartmentalized within the upper respiratory tract (URT) mucosa, or that more subtle functional defects in pneumococcal-specific T cell memory and regulation occur early in the evolution of HIV disease.

In this study conducted in an African population, we show that in asymptomatic HIV infection (WHO stage I) there is considerable immune activation with increased proportions of senescent and Treg cells. We find that, even where there is high pneumococcal exposure, pneumococcal-specific effector memory interferon–gamma (IFN-γ) responses are decreased and that replenishment of this pool may be hindered by poor proliferative capacity of pneumococcal specific central memory (T_CM_) T cells. These defects are associated with a shift in the balance of interleukin (IL)-17 and IFN-γ responses, and failure of activated T cells to express the co-stimulatory molecule CD154 showing that circulating CD4 T cells already show high degrees of immune dysfunction.

## Materials and Methods

### Ethical approval

The collection of samples and the research described was approved by The College of Medicine Research Ethics Committee, Malawi (P.03/08/626, P.01/09/717) and The Liverpool School of Tropical Medicine Research Ethics Committee (08.41, 08.61).

### Subjects

85 adults were recruited from the voluntary counseling and testing clinic (VCT) at Queen Elizabeth Central hospital in Blantyre, Malawi following written informed consent. Of the 85 subjects; 31 were healthy controls confirmed by two HIV rapid antibody tests Uni-gold (Trinity Biotech plc, Bary, Ireland) and Determine (Abbott Laboratories, Minato-ku, Tokyo Japan) and 54 subjects were asymptomatic HIV-infected adults (WHO stage 1). Peripheral blood CD4 T cell counts were determined by flow cytometry using FacsCount (Becton Dickinson, Oxford, UK).

### Assessment of pneumococcal colonization

Nasopharyngeal swabs were obtained and immediately placed into a vial of skim milk-tryptone-glucose-glycerol (STGG) transport medium[8]. When required these were thawed and plated on gentamicin blood agar for the isolation of *S. pneumoniae*. Pneumococci were identified by morphologic characteristics and optochin susceptibility.

### Antigens

Pneumococcal culture supernatants were prepared from a standard encapsulated type 2 (D39) *S. pneumonia* strain and an isogenic pneumolysin-deficient mutant (Ply-; was kindly provided by James Paton)[17]. Bradford protein assay was used to measure the concentration of the concentrated pneumococcal culture supernatant (CCS). The concentrated culture supernatants were heat-inactivated at 56°C for 30 min to reduce toxic effects of pneumococcal proteins (in the absence of heat inactivation toxicity frequently inhibited proliferative capacity). *M. tuberculosis* purified protein derivative (PPD RT49) was obtained from Statens Serum Institut. Influenza antigens were derived from dialyzed inactive trivalent split virion influenza vaccine (Enzira®2006/2007) obtained from Aventis-Pasteur. Phytohemagglutinin (PHA), phorbol 12-myristate 13-acetate (PMA) and ionomycin were obtained from Sigma-Aldrich, St-Louis, MO.

### Cells and reagents

Peripheral blood mononuclear cells (PBMC) were isolated from blood by 25 min centrifugation at 400 g on a density-gradient (Histopaque, Sigma). PBMC were harvested, washed in hanks balanced salt solution (HBSS, Invitrogen, Paisley, UK) at 400 g for 10 min, and resuspended in complete RPMI (RPMI-1640 with 100 U/ml penicillin, 0.1 mg/ml streptomycin, 4 mM _L_L-glutamine and 10 mM HEPES buffer). PBMC were counted using 0.4% (wt/vol) trypan blue (Sigma), reconstituted in complete RPMI at a concentration of 1×10^6^cells/ml; with a final concentration of 2% (vol/vol) heat-inactivated human AB serum (National Blood Services, Blantyre) and incubated at 37°C in presence of 5% CO_2._

### Phenotyping of T cell subsets

20 µl of whole blood was stained at room temperature in the dark for 10min with differing combinations of specific antibodies conjugated to selected fluorochromes: CD3, CD38 and CD25 conjugated to fluorescein isothiocyanate (FITC), CD4, CD8 conjugated to peridinin chlorophyll protein (Percp), CD45RA phycoerythrin (PE), CCR7 allophycocyanin (APC) (all from BD Bioscience, San Jose, CA), PD1-PE and Foxp3-PE (eBioscience, San Diego, CA). After surface staining, red blood cells were lysed with 1X Facs lysing solution (BD). Intracellular staining for Foxp3 was performed after fixation and permeabilization with Foxp3 staining buffer set as per manufactures instructions (eBioscience). For all flow cytometric assays, 10,000–60,000 events were acquired within the lymphocyte gate on either FACSCalibur (BD Biosciences, UK) or CyAn ADP 9 Color (Beckman Coulter, USA) flow cytometers. Analysis was performed with FlowJo (TreeStar, Ashland, OR).

### Ex vivo enzyme-linked immunosorbent spot (ELISPOT) assay

Freshly isolated PBMC were assayed for cells producing IFN-γ, using an ELISPOT assay as previously described[8]. SFC were quantified with an automated ELISPOT reader (AID), and data were expressed as SFC per million PBMC.

### Proliferation assay

Peripheral blood mononuclear cells were labeled with 2.5 µm carboxyfluorescein diacetate 5,6 succinimidyl ester (CFSE) dye (Invitrogen) to identify the dividing T cell population. Labeled cells were cultured at 1×10^6^cells/ml in a 48-well plate with WT CCS (8 µg/ml), Ply-CCS (8 µg/ml), PPD (10 µg/ml), influenza (0.9 µg/ml), PHA (10 µg/ml) or media only, for 8 days. Cells were harvested and stained with anti-CD4-APC (BD Biosciences) and assessed by flow cytometry. Antigen specific T cell proliferation was expressed as the percentage of CD4^+^cells. In a subset of individuals, PBMC were further stimulated with PMA (100 ng/ml) and ionomycin (500 ng/ml) on day 8 for 6h and pneumococcal specific CFSE_low_ proliferating cells were evaluated for IFN-γ and IL-17 production using intracellular cytokine staining. Brefeldin A (10 µg/ml) (Sigma) was added 1h after polyclonal stimulation to block the secretion of cytokines. Cells were harvested and stained with anti-CD4-PerCp, CD8-ECD (Beckman Coulter) and CD3-APC-H7 (BD Bioscience) for 10 min. After washing with PBS, cells were permeabilized and fixed using cytofix/cytoperm (BD Bioscience) as per manufactures instructions. Cells were then incubated for 30 min at 4°C with anti-IFN-γ-APC and IL-17-PE (BD Bioscience) antibodies. Cells were washed with 1x Perm Wash (BD Bioscience), resuspended and analyzed by flow cytometry.

### Detection CD154 expression as measured by surface mobilization assay

Total PBMC 1.5–2×10^6^ were resuspended in 200 µl complete RPMI and plated in a 96-well plate. Anti-CD154-PE antibody was added prior to antigen stimulation to capture the surface and transient expression of CD154 as previously described[18]. The cells were stimulated with WT CCS (8 µg/ml), Ply-CCS (8 µg/ml), *M. tuberculosis* PPD (4 µg/ml), PHA (10 µg/ml) or media only for 16hs. After stimulation, cells were harvested, washed and surface-stained with anti-CD3-FITC, CD4-PerCP, CD69-APC and CD154-PE (BD Biosciences) for 15min at 4°C in the dark. Cells were washed with 1ml PBS at 400g for 5 minutes, resuspended and analyzed by flow cytometry

### Cytokine profiles in cell culture supernatants

Cell culture supernatants were analyzed for IL-10, IL-2, tumor necrosis factor–alpha (TNF-α), and IFN-γ, using the human bioplex cytokine assay (Bio-Rad, Hercules, CA, USA) according to the manufacturer's instructions. Beads were read on the Bio-Plex 100 suspension array system (Applied Cytometry), and data were analyzed using Bio-Plex Manager software, version 3.0. The concentration of IL-17A was measured by IL-17A ELISA (eBioscience), read on Opsys MR plate-reader and analyzed using Revelation QuickLink software (Dynex Technologies, Virginia, USA).

### Statistical analysis

Statistical analyses and graphical presentation were performed using Graphpad Prism 5 (Graphpad). Non-parametric data were analyzed using the Mann-Whitney U test. Results are given as means with standard error of the mean (for parametric tests) or as medians with ranges (for non-parametric tests). Differences were considered significant if *P*≤0.05.

## Results

### Demographic characteristics

31 healthy adult controls (median age 33years [range 23–53]) and 54 asymptomatic WHO stage 1 HIV-infected adults (median age 31years [range 18–58]) were recruited (Table.1). All participants were anti-retroviral therapy (ART) and co-trimoxazole naive, and had no recent history of severe respiratory infection or TB. Healthy controls and those infected with HIV were similar in age, sex distribution and prevalence of pneumococcal carriage. The median CD4 count amongst the HIV-infected participants was 252 (range 7-913 cells/µl). For analysis purposes, HIV infected individuals were stratified by peripheral CD4 count (CD4≥500, CD4 350–500 and CD4≤350cells/µl). WHO currently recommends the commencement of ART when CD4<350cells/µl but this had not been implemented in Malawi at the time of the study[19].

**Table 1:** Patient demographics, age, sex, median CD4 count cells/µl blood, *S. pneumoniae* carriage, WHO clinical stage.

	HIV-n = 31	HIV+n = 54	HIV+ ≥500n = 10	HIV+ 350-500n = 14	HIV+ ≤350n = 30
**Age**	33 (23–53)	31 (18–58)	29 (21–47)	28 (23–40)	32 (18–58)
**Female**	32%	46%	87%	50%	24%
**Male**	68%	54%	13%	50%	76%
**CD4 count (cells/µl)**	853	*252	695	403	192
**CD4 range (cells/µl)**	(444–1323)	(7–913)	(549–913)	(352–495)	(7–292)
**Carriage**	16%	14%	13%	14%	13%
**WHO Stage**	n/a	Stage 1	Stage 1	Stage 1	Stage 1

Not available (NA). *Significance determined by Mann Whitney U, p = ≤0.05 (HIV- vs HIV+).

### Effector memory, senescent and regulatory CD4 T cells in asymptomatic HIV infected Malawian adults

HIV infection is associated with chronic activation of the immune system[20] with an early depletion of memory T cells[21], increased senescent CD4 T cells expressing programmed death 1 (PD-1)[22] and mucosal Tregs[23]. In contrast to industrialized countries, individuals in a resource-poor setting are under increased immune pressure from parasitic diseases such as malaria and micronutrient deficiencies[24], [25]. We therefore investigated the degree of immune disruption caused by HIV in a population of HIV-infected African adults.

We compared the proportional balance of T_EM_ (CD4^+^CCR7^−^CD45RA^−^),T_CM_ (CD4^+^CCR7^+^CD45RA^−^), naïve T cells, regulatory (CD4^+^CD25^high^Foxp3^+^) and senescent (CD4^+^CD38^+^PD-1^+^) T cells in peripheral blood of healthy controls and HIV-infected individuals[26].

**Figure 1:**
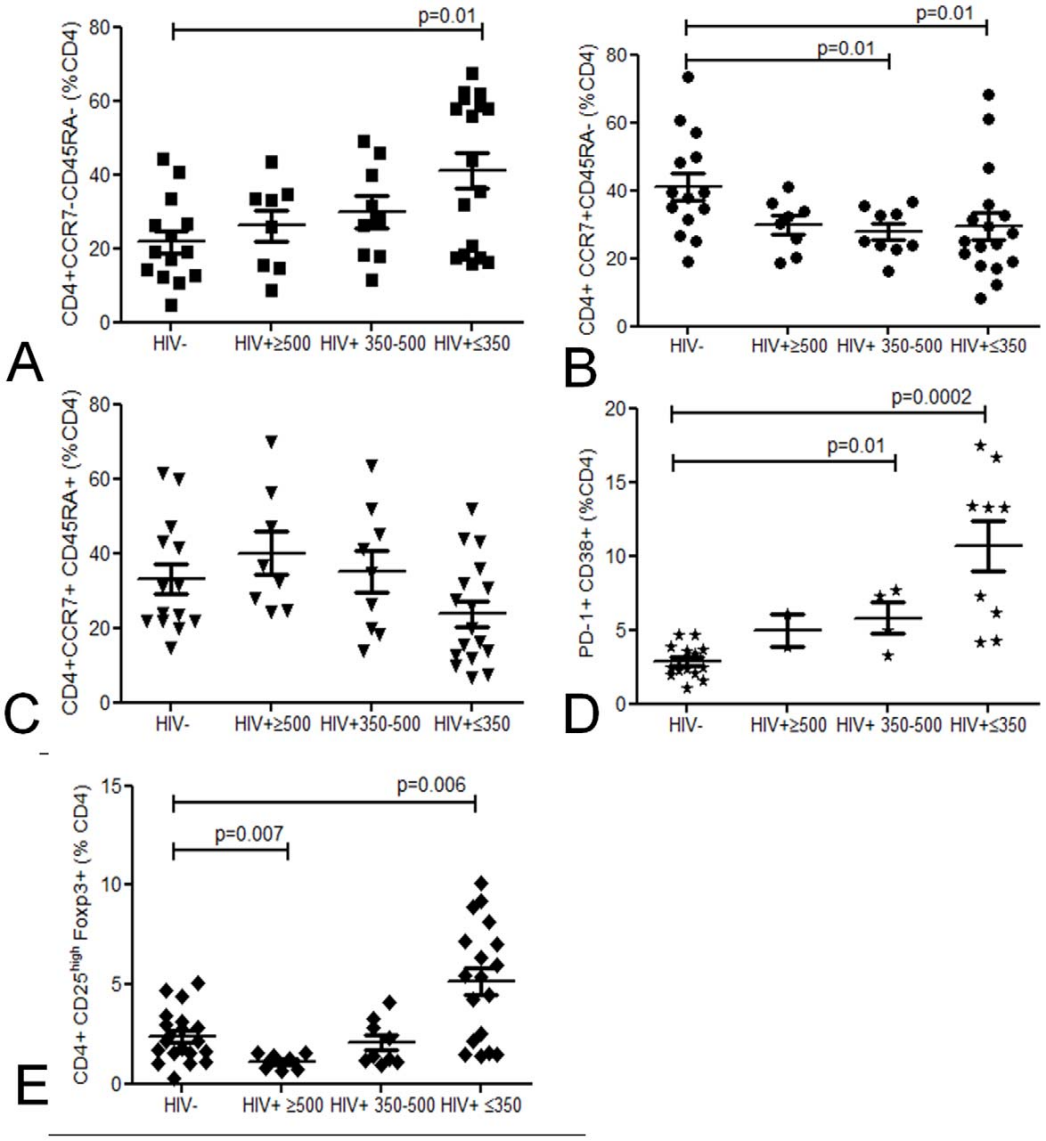
Baseline phenotyping of peripheral blood CD4 T cells. Proportional representation of circulating T_EM_ (CD4^+^CCR7^−^CD45RA^−^) (**a**), T_CM_ (CD4^+^CCR7^+^CD45RA^−^) (**b**), naïve T cells (CD4^+^CCR7^+^CD45RA^+^) (**c**), regulatory (CD4^+^CD25^high^Foxp3^+^) (**d**) and senescent (CD4^+^CD38^+^PD-1^+^) (**e**) CD4 T cells using flow cytometry in healthy controls (n = 8–14) and HIV-infected groups (≥500 (n = 2–10) 350−500 (n = 4-9) ≤350 (n = 17–19) CD4 T cells/µl blood). Black horizontal bars represent median values of CD4 subsets. Statistical significance was analyzed by the Mann Whitney U test.

*Ex vivo* flow cytometric analysis showed a decrease in absolute number of T_CM_ and naive cells in individuals with robust CD4 counts 350-500cells/µl. In contrast, T_EM_ were depleted only in those with very low CD4 counts ≤350cells/µl (data not shown). The preferential depletion of T_CM_ resulted in an imbalance in the proportion of circulating T_EM_ (CD4^+^CCR7^−^CD45RA^−^) vs. T_CM_ (CD4^+^CCR7^+^CD45RA^−^) cells in HIV-infected adults (**Fig. 1ab**). The proportion of naive CD4^+^CCR7^+^CD45RA^+^ T cells was comparable between healthy controls and HIV-infected individuals (**Fig. 1c**). This relative increase in T_EM_ cells was coupled with an increase in CD4^+^CD38^+^ T cells expressing the co-inhibitory molecule PD-1, which is associated with immune activation and senescence in both naïve and memory T cells[27](**Fig. 1d**). These findings show that HIV infection drives persistent immune activation[28] and eventual exhaustion early on in asymptomatic HIV-infection in a African population. Furthermore, CD4^+^CD25^high^Foxp3^+^ Treg were proportionally increased in those infected with HIV (**Fig. 1e**) thus altering the ratio of Treg: T cell responders.

### Antigen-specific IFN-γ effector responses in HIV infection

**Figure 2:**
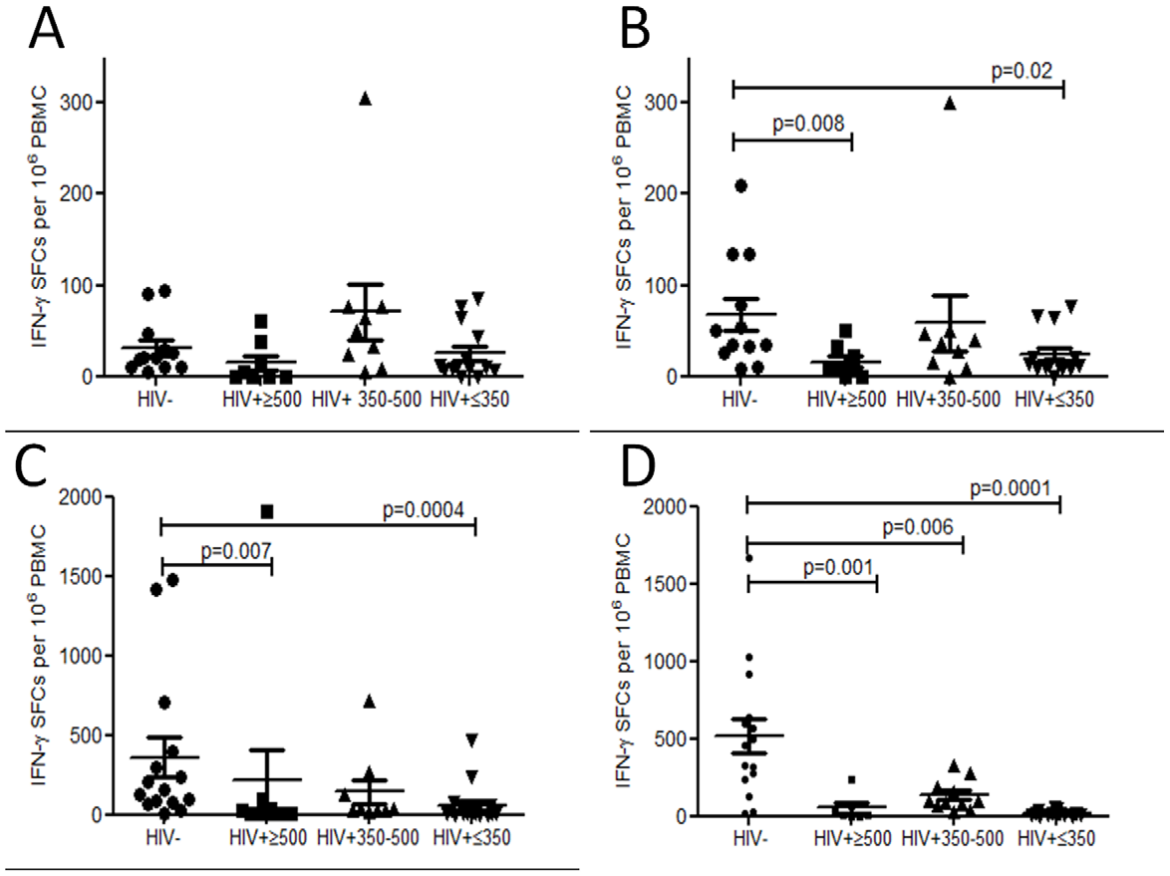
Antigen-specific *ex vivo* IFN-γ responses. (**a**) in response to a wild-type *Streptococcus pneumoniae* strain (WT), (**b**) an isogenic pneumolysin (Ply)–deficient mutant (Ply-), (**c**) *M. tuberculosis* PPD and (**d**) influenza for 16 hours by IFN-γ enzyme-linked immunosorbent spot (ELISPOT) stimulation. Collated data from controls (n = 12–15) and those infected with HIV (≥500 (n = 7–10) 350-500 (n = 9–11), ≤350 (n = 14–17) CD4 T cells/µl). Black horizontal bars represent median values of total antigen specific responses above background. Statistical significance was analyzed by the Mann Whitney U test.

HIV targets recently activated mucosal effector cells in the gut early on in disease, partly due to their high expression of HIV co-receptors[15], [29]. To evaluate whether HIV infection leads to antigen-specific depletion of peripheral effector memory generated within the URT, pneumococcal T_EM_ IFN-γ responses were evaluated in comparison to other respiratory pathogens, *M. tuberculosis* and influenza (not typically associated with prolonged asymptomatic mucosal carriage). The number of IFN-γ producing cells responding to WT CCS was low but detectable in healthy controls and was relatively well preserved in HIV infection (**Fig. 2a**). To assess the potential immunomodulatory effect of pneumolysin[30], Ply^−^CCS was used to stimulate IFN-γ responses. In healthy controls, there were more IFN-γ producing cells in response to Ply^−^CCS as compared to WT CCS but a decreased pneumococcal IFN-γ response was seen in HIV-infected individuals (**Fig. 2b**). IFN-γ producing cells were also decreased in response to *M. tuberculosis* PPD (**Fig. 2c**) and influenza antigens (**Fig. 2d**) in HIV-infected persons. We have previously shown that that *ex vivo* IFN-γ pneumococcal responses are derived from CD4^+^CD45RO^+^CCR7^−^ effector memory cells[8], thus a lack of IFN-γ responses reflects either the depletion or an impairment of T_EM_ function.

### HIV infection impairs antigen-specific CD4 T cell proliferation

**Figure 3:**
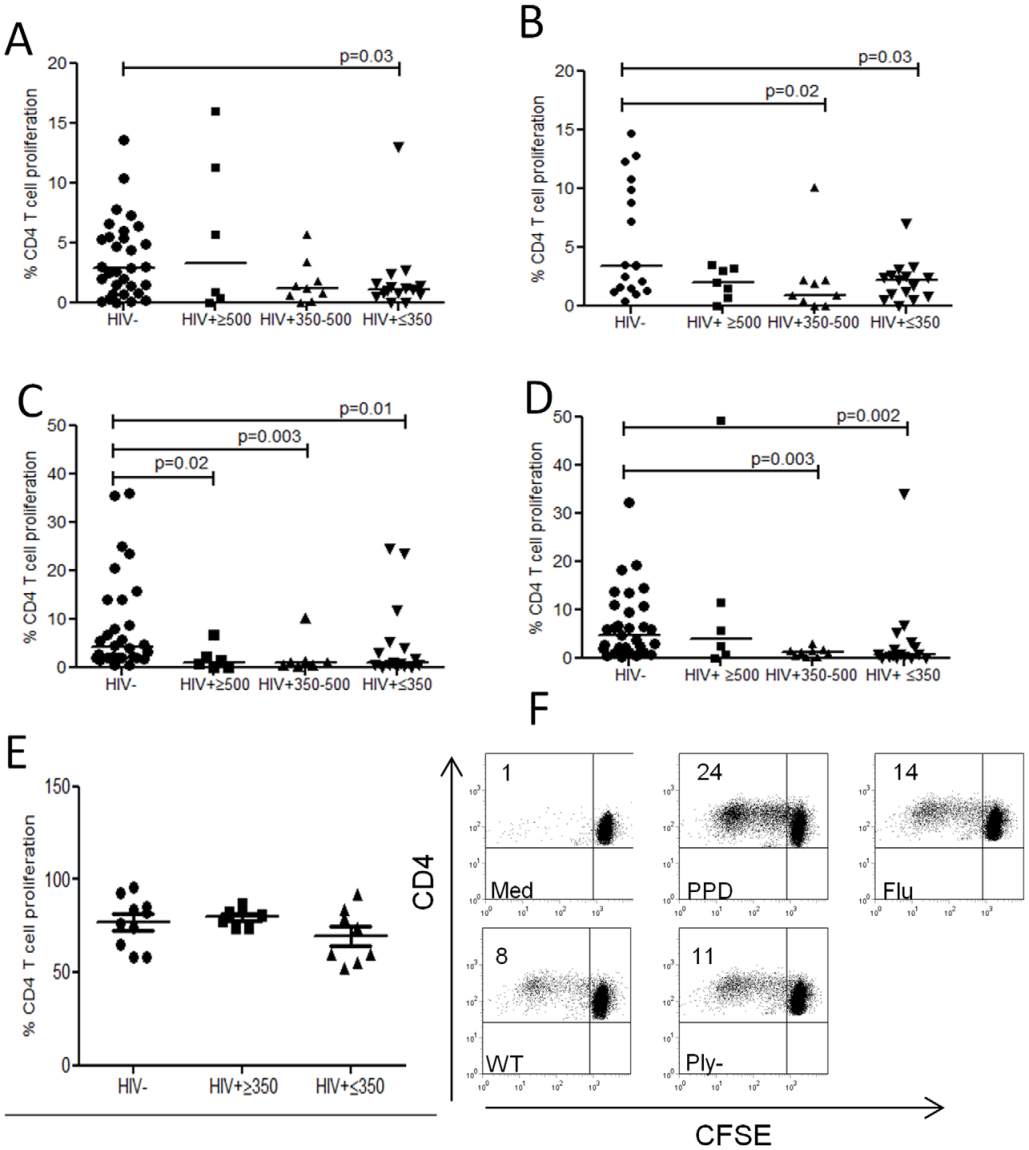
Proliferative capacity of antigen–specific CD4+ T cells. (**a**) in response to a wild-type *Streptococcus pneumoniae* strain (WT), (**b**)an isogenic pneumolysin (Ply)–deficient mutant (Ply_), (**c**) M. tuberculosis PPD (**d**) influenza (**e**) phytohemagglutinin (PHA) using flow cytometry. Collated data from controls (n = 18–31) and those infected with HIV (≥500 (n = 6−7), 350–500 (n = 7−9), ≤350 (n = 15−16) CD4 T cells/µl blood). Black horizontal bars represent median values of total antigen specific responses above background. Statistical significance was analyzed by the Mann Whitney U test. (**f**) Representative flow cytometric data demonstrating CD4+ T cell proliferative responses in media only, *M. tuberculosis* PPD, influenza and pneumococcal antigens. The percentage indicated in the upper-left quadrant represents the cell population that proliferated over the 8-day culture period.

To reconstitute the antigen specific T_EM_ pool, T_CM_ must rapidly expand, differentiate and migrate to effector tissue sites[26]. We therefore evaluated the proliferative capacity of T cells in response to WT, Ply^−^ CCS, *M. tuberculosis* PPD, influenza antigens and the polyclonal stimulus PHA. In HIV infection, we found impaired proliferation in response to WT CCS (**Fig. 3a**), Ply^−^CCS (**Fig. 3b**), *M. tuberculosis* PPD (**Fig. 3c**) and influenza (**Fig. 3d**) compared to healthy controls, whilst intrinsic proliferative capacity to PHA remained intact (**Fig. 3e**). These data suggest that the cognate interaction required for the renewal or maintenance of antigen-specific CD4 proliferating T cells is impaired.

### Failure to upregulate CD154 (CD40L) on activated CD4 T cells

**Figure 4:**
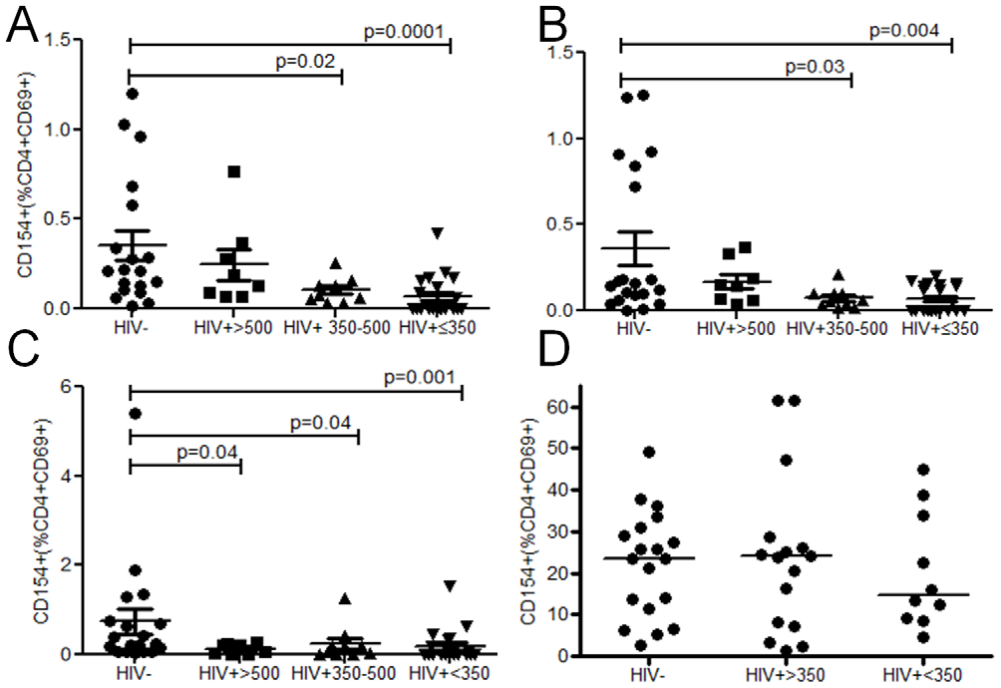
CD154 expression on CD4+CD69+ T cells. (**a**) in response to a wild-type *Streptococcus pneumoniae* strain (WT), (**b**) an isogenic pneumolysin (Ply)–deficient mutant (Ply_), (**c**) M. tuberculosis PPD and (**d**) phytohemagglutinin (PHA) in HIV- controls (n = 19-20) and those infected with HIV (≥500 (n = 8), 350–500 (n = 10), ≤350 (n = 21) CD4 T cells/µl blood). Black horizontal bars represent median values of total antigen specific responses above background. Statistical significance was analyzed by the Mann Whitney U test.

CD40 dependent impairment of CD4 T cell CD154 expression has been described in HIV infection[31] and reduced CD40L-CD40 interaction may contribute to poor pneumococcal-specific T cell proliferation and/or effector function. We therefore evaluated whether recently activated CD4^+^CD69^+^ T cells responding specifically to pneumococcal and tuberculin antigens were capable of upregulating this critical co-stimulatory molecule. In HIV infection, T cell activation (CD4^+^CD69^+^) was reduced in response to *M. tuberculosis* PPD but not in response to the pneumococcus (data not shown). Nevertheless the expression of CD154 on activated T cells was impaired in response to WT, Ply^−^CCS and *M. tuberculosis* PPD suggesting a specific decrease in CD154 regulation that did not necessarily follow a generalized decrease in cellular activation and CD69 upregulation (**Fig. 4**). The finding that CD154 was expressed in response to PHA (which activates T cells in the absence of CD40^+^APC[32]) (**Fig. 4d**) shows that the defect is not intrinsic to the T cell, as suggested by others[33], but is likely to occur during a cognate interaction with APCs.

### Preservation of pneumococcal-specific IL-17 but impairment of IFN-γ production

**Figure 5:**
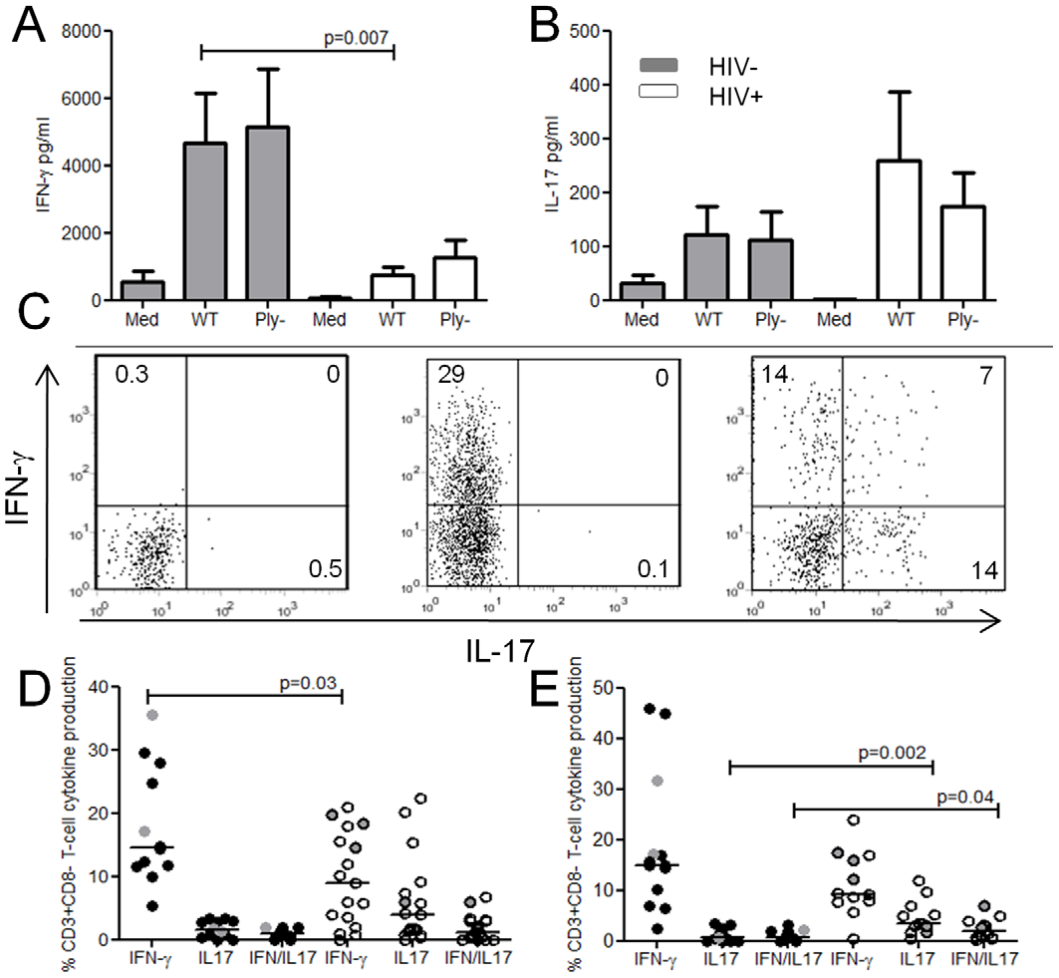
IFN-γ and IL-17 production. Concentration of (**a**) IFN-γ, and (**b**) IL-17 in PBMC cell culture supernatants stimulated with wild-type *Streptococcus pneumoniae* strain (WT), and an isogenic pneumolysin (Ply)-deficient mutant and media only from healthy controls (grey bars) (n = 6) and HIV-infected subjects (white bars) (n = 9). Data are mean values ± standard error of the mean. (**c**) Representative flow cytometric data of media only, HIV- and HIV+ T cells producing IFN-γ and IL-17 following WT CCS stimulation. CFSE_low_ pneumococcal-specific proliferating T cells were selectively gated and cytokines evaluated. (**d**) CFSE_low_ pneumococcal specific T cell IFN-γ and IL-17 production following WT and (**e**) Ply-CCS in HIV- (black circles n = 11) and HIV+ (white circles n = 13−17) individuals. Grey data points represent pneumococcal carriers (nasopharynx). Black horizontal bars represent median values of total antigen specific responses above background. Statistical significance was analyzed by the Mann Whitney U test.

Th1 and Th17 cells have been shown to be important in facilitating the clearance of the pneumococcus[11], [12] and controlling multiplication of bacteria following dissemination[13], [14]. We have suggested that these responses are moderated by Treg in healthy populations[34]. Th1 cells classically produce IFN-γ, TNF-α and IL-2, Th17 cells produce IL-17 and a subset of Treg produce IL-10[35]. To investigate whether an increase in IPD risk is associated with a selective loss of either Th17 or Th1subsets or the over-representation of Tregs, we investigated the balance of these cytokines in cell culture supernatants from pneumococcal 8 day stimulation assays. In healthy controls, stimulation with WT and Ply^−^CCS resulted in the production of IFN-γ (**Fig. 5a**) and IL-17 (**Fig. 5b**). In HIV infection, IFN-γ production was greatly reduced, whilst IL-17 production was not significantly different to healthy controls. Concentrations of IL-10, TNF-α, and IL-2 were similar between HIV-infected individuals and healthy controls (data not shown). As cytokine concentrations are influenced by the number and proliferative capacity of cells in culture, we evaluated intracellular IFN-γ and IL-17 from proliferating pneumococcal-specific T cells using flowcytometry(**Fig. 5c**). In HIV infection, proportionally less pneumococcal-specific T cells produced IFN-γ as compared to healthy controls whereas IL-17 and IFN-γ/IL-17 double positive responses were increased(**Fig. 5d&e**). In contrast, influenza and *M. tuberculosis-*specific T cells produced no IL-17 but IFN-γ was consistently decreased in those infected with HIV (data not shown).

## Discussion

Invasive pneumococcal disease remains an important cause of HIV-related death in Sub-Saharan Africa (SSA), even in the era of widespread ART roll-out[36]. For the effective design and evaluation of both existing and novel pneumococcal vaccines for implementation in SSA adult populations, pneumococcal immunity must be studied where disease burden is greatest and where poor nutrition and parasitic infections such as malaria compound disease susceptibility in HIV-infected individuals[24]. Our investigations have therefore targeted African adults with asymptomatic HIV infection and relatively preserved CD4 counts to identify which immunological processes are affected early in the progression of the disease. We show considerable immune dysfunction with a skew towards T_EM_ and activated CD4 T cells expressing the co-inhibitory senescence marker PD-1. In the context of this immune activation and exhaustion, we show reduced pneumococcal-specific IFN-γ effector responses and impaired T_CM_ proliferative function, thus limiting the capacity to replenish the effector pool. Whilst IL-17 responses appeared to be preserved in asymptomatic HIV infection, IFN-γ production was greatly reduced in dividing T cells. Furthermore, pneumococcal-specific activated T cells failed to upregulate CD154 (CD40L), a key mediator of T cell- B cell/APC cross talk[37]. Together these data implicate defects in immune regulation, T-helper function and T-B cell signaling with a shift in the normal balance of Th1 and Th17 cells in the early phases of disease progression. Factors such as poor epithelial barrier integrity[38] and reduced innate antimicrobial defensins[39] may further exacerbate this immune imbalance[24].

Given the intensity of pneumococcal colonization in African populations[8] and our data showing localized anti-pneumococcal mucosal immune responses[7], we had expected that activated pneumococcal-specific cells would be preferentially depleted by HIV, possibly through HIV co-receptors such as the mucosal homing receptor α4β7[29] or CD25 expression which is linked to enhanced HIV replication and cellular apoptosis[40]. Interestingly, we found no evidence for selective depletion of pneumococcal-specific T cells when compared with immunity to *M. tuberculosis* and influenza virus, respiratory pathogens not typically associated with commensalism at the mucosal surface. There are several potential explanations for this apparently more generalized memory T cell depletion, including mucosal compartmentalization of pneumococcal-specific T cell depletion and HIV infection of all resting memory T cells regardless of specificity[41]. We suggest that even though antigen exposure is less common, the inflammatory nature of the immune response to influenza and mycobacteria produces a high frequency of central memory T cells which are prone to HIV infection and depletion. In fact, Geldmacher et al have recently demonstrated that TB-specific CD4 T_CM_ expressing IL-2 were preferentially depleted early on in HIV infection (6−12 months following HIV seroconversion) as compared to CMV-specific CD4 T cells[42]. In contrast, the pneumococcus, a highly adapted commensal, avoids activation of adaptive immunity at the mucosal surface by evading recognition, inducing tolerance or regulation[43], [44]. Thus, although encounter is frequent, pneumococcal-specific T cells are less vigorously targeted for HIV-mediated depletion.

Murine models have demonstrated that effective clearance of colonizing pneumococci requires a population of Th17 cells and the recruitment of neutrophils[11], [12]. Potentially a preferential loss of Th17 cells in the URT could cause an increase in the frequency and/or intensity of pneumococcal colonization[12] and thus increase the risk of bacterial dissemination. Interestingly, we find no increase in pneumococcal carriage in asymptomatic Malawian adults with HIV infection and show that despite other antigen-specific defects in T cell memory, IL-17 production to pneumococcal antigens is preserved, at least in the periphery. However, our data does raise the alternative possibility that although control of pneumococcal colonization is unaffected in this population, the risk of disease is increased because of failure to control pneumococcal invasion and dissemination following colonization, in part due to impaired IFN-γ production. Our findings show that T cells producing IFN-γ decline before those that produce IL-17 thereby severely disabling arms of the immune response that are enhanced by IFN-γ, such as B cell isotype switching and macrophage function[13], [14]. We propose that poor T cell IFN-γ responses coupled with defective pneumococcal-specific CD154 expression fails to enhance macrophage phagocytosis and microbial killing when recruited to sites of tissue invasion. Furthermore, CD154 deficient T cells homing to B cell follicles will fail to facilitate B cell production of pneumococcal-specific antibodies[45].

Several studies have shown that the induction of CD154 is decreased in HIV-1-infected individuals and it is noteworthy that chimpanzees that are susceptible to chronic infection by HIV-1 but rarely develop subsequent immunodeficiency or disease progression, retain their ability to upregulate CD154 in the context of HIV[46]. In keeping with previous reports[47], we have found that cross-linking of CD4 with a monoclonal antibody to domain 1 also leads to inhibition of CD154 upregulation in response to pneumococcal antigens (data not shown). We therefore postulate that cross-linking of CD4 by HIV glycoproteins can disrupt normal T cell signal transduction in the early stages of HIV infection when viral load is high, thus providing another mechanism for the impairment of CD154. Furthermore, the proportional increase in regulatory T cells and an over-representation of CD4^+^CD38^+^PD-1^+^ T cells which are almost certainly HIV-specific, may contribute to impaired effector IFN-γ production, T cell proliferation and T cell CD40L expression, by indirectly perturbing DC function[23], [48].

Demonstration of immune dysregulation of anti-pneumococcal immunity even in patients with CD4 counts that exceed the current threshold for commencing ART (<350cells/µl), coupled with high pneumococcal exposure and therefore immune stimulation in HIV-infected Malawian adults has direct implications for the design and implementation of both ART and protective vaccines. Early introduction of ART not only restores T cell numbers but also improves T cell function[49]. It remains to be determined whether the early T cell recovery results in a reversal of the multiple T cell defects we observed and restores a balanced pneumococcal specific-Th1:Th17 response. In view of the encouraging efficacy of the pneumococcal conjugate vaccines in high risk HIV adults[50] but their reliance on carrier protein-induced T cell help, it will be necessary to assess whether this success can be translated into long-term immune protection against IPD or whether defects affecting T cell function and regulation of immunity to the carrier protein leads to poorly sustained immune memory.
